# Dietary Diversity and Associated Factors among Pregnant Women Attending Antenatal Clinic in Shashemane, Oromia, Central Ethiopia: A Cross-Sectional Study

**DOI:** 10.1155/2019/3916864

**Published:** 2019-03-12

**Authors:** Melaku Desta, Mohammed Akibu, Mesfin Tadese, Meskerem Tesfaye

**Affiliations:** ^1^Debre Markos University, College of Health Science, Department of Midwifery, Debre Markos, Ethiopia; ^2^Debre Berhan University, Institute of Medicine and Health Science, Department of Midwifery, Debre Berhan, Ethiopia; ^3^Hawassa University, College of Health Science, Department of Midwifery, Awasa, Ethiopia

## Abstract

**Background:**

Maternal dietary diversity is a proxy indicator of maternal nutrient adequacy and improves health outcomes for both mothers and babies. However, little is documented on dietary diversity among pregnant mothers. Therefore, this study assessed diet diversity and associated factors among pregnant mothers attending the antenatal clinic in Shashemane, Oromia, Central Ethiopia.

**Methods:**

An institution-based cross-sectional study was conducted on 315 systematically selected pregnant women attending antenatal clinic of Shashemane town in April 2017. Dietary diversity was assessed using a 24 h dietary recall method, and the dietary diversity score was computed for ten food groups. Bivariate and multivariate logistic regressions were computed to identify associated factors of dietary diversity.

**Result:**

In this study, only a quarter (25.4%) of pregnant mothers consumed adequate dietary diversity. Mother's tertiary (AOR 3.18; 95% CI: 1.8, 6.35) and secondary (AOR 2.13; 95% CI: 2.32, 8.72) education, household monthly income above 3500 ETB (AOR = 2.24; 95% CI: 1.47, 7.78), livestock ownership (AOR = 4.15; 95% CI: 2.07, 9.86), women who got emotional support from the husband (AOR = 3.49; 95% CI: 1.12, 8.23), and women who participated in the shooping (AOR = 2.54; 95% CI: 3.27, 9.83) were more likely to attain the adequate dietary diversity.

**Conclusion:**

The study revealed that the overall consumption of adequate dietary diversity was found to be low. Developing the educational level of women, increasing household income and owning of livestock, increasing husbands' support, and improving women's participation in the shopping are recommended to improve women's adequate dietary diversity.

## 1. Introduction

Multiple micronutrient deficiencies remain a major public health concern in low-income and middle-income countries (LMICs) especially among reproductive women. Pregnancy places an additional burden on women's nutritional requirement, as nutrient needs increase to meet the demands of both the mother and the developing fetus [1]. Malnutrition during pregnancy can permanently affect physiological development of the fetus, increases risk of intrauterine growth restriction, low birth weight, preterm delivery, and maternal morbidity and mortality [2].

Adequate nutrient intake during pregnancy was found to reduce the risk for low birth weight (19%), small-for-gestational-age births (8%), preterm birth by 16%, and infant mortality by 15% in those highly adhered to the regimen. In underweight women, multiple micronutrient supplementation initiation before 20 weeks' gestation decreased the risk of preterm birth by 11% [3, 4].

Antenatal healthy diet initiation in pregnancy and high adherence to multiple micronutrients supplements also provided greater overall benefits [5, 6]. Dietary diversification is one of the best strategies highly recommended among pregnant women which associated with improved diet adequacy through increased food groups in daily diet. Dietary diversity has been defined as the number of different food groups that are consumed over a specific reference period [5, 7–10].

The burden of nutritional deficiency among pregnant women is still high in sub-Saharan Africa [11–14]. Even though dietary diversification is essential for the health of the mother and the fetus in Ethiopia, 22% of women are thin and 8% of women are obese due to inadequate dietary intake, limited diet diversity (vegetables and fruits), and changing lifestyles [15].

Studies have revealed that the global mortality and morbidity due to malnutrition has not significantly changed over the last 30 years [11, 13]. In order to improve it, dietary diversification status of pregnant women needs to be sustainably improved through addressing of information and reduction of barriers of adherence. However, there is no study in Ethiopia in the study area of interest. Therefore, this study was aimed to assess dietary diversity and associated factors among pregnant women visiting public health facility in Shashemane town, Central Ethiopia. The finding of this study will help policymakers and planners to set their target with interventions in the study area and might be a means of achieving the sustainable development goals (SDGs) in an integrated manner.

## 2. Methods and Materials

### 2.1. Study Design and Setting

An institution-based cross-sectional study was conducted in Shashemane, found in West Arsi Zone Oromia regional states, and it is also the capital of the woreda. It is 250 km far from Addis Ababa and 25 km far from Hawassa. Bulchana is one of the kebelle (smallest administrative unit in Ethiopia) in Shashemane having a total of 50,500 population of whom, 9417 were reproductive age mothers and from this reproductive age, 1754 of them were pregnant and 670 pregnant women had antenatal care (ANC) visit in Bulchana Healthcenter [16].

### 2.2. Participants

The source population was pregnant mothers who have ANC follow-up in Bulchana Healthcenter, and the sample populations were the selected mothers who have ANC visit to participate in the study period. All pregnant women who consented to participate in the study and who lived in Shashemane for at least one year prior to the period of the study were included. One year was appropriate as it covered the whole food security cycle. Pregnant women with chronic diseases such as cancer and diabetes were excluded from the study. Hence, these conditions are known to have impact on food intake, the nutritional status of an individual, and those who were unable to communicate due to illness (hyperemesis gravidarum and preeclampsia).

### 2.3. Sample Size and Sampling Procedure

The required sample size was determined by single proportion formula based on the assumption of the recommended minimum dietary diversity of 85% and educational status as determinants in Kenya [17], 5% marginal error, 95% CI, and a nonresponse rate of 10%. The final sample size was 329, and systematic random sampling was used to select the participants. The interview was started by selecting a random sample, and then, every 2nd pregnant women were included.

### 2.4. Variables and Measurement

The dependent variable of the study was the minimum dietary diversity, and the independent variables were maternal factors (age, educational level, occupation, marital status, parity, morbidity, and participation in shopping), household factors (household asset, land ownership, family size, family's income, emotional support by the husband), and number of ANC visits. Dietary diversity was coded as 1 for those meeting the minimum dietary diversity and 0 for those not achieving minimum dietary diversity.

*Dietary diversity score* is the number of food groups consumed by pregnant women out of the ten food groups. These food groups include starch staples, vitamin A-rich vegetables and fruits; dark green leafy vegetables; other vegetables; other fruits; meat, poultry, and fish; eggs and pulses/legumes; nuts and seeds; and dairy products.

*Minimum dietary diversity* is consumption of five or more food groups of the fourteen food groups used in this study [10].

*Adequate dietary diversity* represents those women meeting the minimum dietary diversity.

*Market participation* represents women involved in the market at least once in the previous one month.

### 2.5. Data Collection Technique and Quality Control

A structured questionnaire was adapted from a validated and modified individual dietary diversity questionnaire as recommended by the Food and Agriculture Organization individual and household dietary diversity guideline (FAO, 2016 [10] and other related literature [17]). A pre-test was done on 5% of the total sample size on other nearby health facilities, and training for 3 days was given. A face-to-face interview questionnaire was prepared in English and translated into the local language and finally back to English. Four diploma midwives were collecting the data, and one senior staff was recruited as supervisor. Dietary diversity of the respondents was assessed by 24 h recall; a single point was given to each food group consumed over the reference period and a sum total of all points was calculated. Cronbatch's alpha was done (*p*=0.83), and the collected data were checked on the daily basis for completeness and consistency.

### 2.6. Data Processing and Analysis

The data were checked, cleared, and entered into Epidata 3.1 software, and analysis was done by using SPSS version (20.0). The dietary diversity score variable was dichotomized as category 1 for those meeting the minimum diversity and 0 for those not meeting the minimum dietary diversity. Bivariate and multivariable logistic regressions were carried out to assess factors of dietary diversity. Model fitness was checked using Hosmer and Lemeshow statistics (*p*=0.45), which showed fitted. Variables with a *p* value less than 0.25 in the univariate analysis were entered into a multivariate logistic regression analysis. Variables with a *p* value < 0.05 were used as statistically significant factors. The analysis was done by reporting of STROBE statement checklist [18].

### 2.7. Ethical Considerations

Ethical clearance and permission were obtained from Hawassa University, and informed consent was obtained and confidentiality was ensured.

## 3. Results

### 3.1. Demographic and Socioeconomic Characteristics

A total of 315 respondents were involved in the study with a response rate of 95.7%. The mean age of the pregnant women was 22.7 ± 4.37 (SD) years, and a majority (77.1%) of the participants were in the age group <25 years. A majority of the respondents (71.4%) were protestant religion followers, and all participants were married. A proportion of women (92.7%) and 299 (94.9%) of their husbands had attended primary and secondary school. Regarding occupation, 129 (41.0%) were housewives and greater than three-fourths of respondents (80.7%) earned less than 2000 Ethiopian birr per month. Regarding household assets, nearly half (47%) of the respondents reported owning livestock and above three-fourths of the participants (77.8%) reported owning a piece of land. Notably, 86.3% of the respondents participated in the market exchange. In regard to household characteristics, 81.6% of household size was 1–3 persons and one-third of the participants got support from their husband for ANC visit and to get the diversified diet in pregnancy. In regard to maternal morbidity, the study showed that 43.8% participants reported some form of illness disorder in the preceding two weeks prior to the interview and heartburn was the commonest disorder reported by 54.5% of the study participants (Table 1).

### 3.2. Source of Food and Feeding Practices during Pregnancy

Concerning the practice, 15 (4.8%) of women follow specific dietary regimen and 52 (16.5%) of women used iodized salt to prepare their daily main meals. Concerning caffeine drinking, greater than nine-tenths (91.1%) of pregnant women had the habit of drinking coffee and tea during meal, right after meal, and right before meal. Regarding the meal frequency per day, only 73 (23.2%) had diet frequency of four and above per day. Less than one-fourth, 64 (20.3%), of respondents had the habit of taking snacks, and concerning micronutrient supply, 26 (8.3%) of women had iron tablets. Notably, the major source of food among the respondents was found to be purchased (52.4%) (Table 2).

### 3.3. Dietary Diversity of Pregnant Women

Out of the 10 food groups, the study found that the mean dietary diversity score among pregnant women was 3.48 ± 2.46 SD with scores ranging from 3 to 7 food groups. Based on the categories developed, only 25.4% (95% CI: 2.6, 7.5) of pregnant women receive minimum dietary diversity. Regarding the consumed food groups by pregnant women in the previous 24 hours, nearly all women (95%) consumed starchy staples, two-thirds of women consumed other vegetables, and 58.7% consumed nuts and seeds. Moreover, foods of the animal product were minimally consumed, one-third consumed (33.3%) dairy products, and the egg was the least consumed (Table 3).

### 3.4. Associated Factors of Dietary Diversity

The association of dependent and independent variables were explored by both bivariate and multivariate binary logistic regressions. The multivariate analysis showed that educational level, family's income, livestock ownership, emotional support by the husband, and market exchange participation were significantly associated factors of adequate dietary diversity (Table 4). Those pregnant women who had tertiary (AOR 3.18; 95% CI: 1.8, 6.35) and secondary education (AOR 2.13; 95% CI: 2.32, 8.72) had three times and two times more likely to achieve the adequate dietary diversity, respectively, as compared to those who had no formal education. The study also showed those pregnant women whose household income was above 3500 ETB (AOR = 2.24, 95% CI: 1.47, 7.78) had greater odds of attaining adequate dietary diversity than those women whose household income was less than 2000 ETB. Similarly, pregnant women who had reported owning livestock had 4 times (AOR = 4.15, 95% CI: 2.07, 9.86) greater chance of achieving adequate dietary diversity when compared with those who did not own. Furthermore, those women who got husbands' support were 3.5 times (AOR = 3.49, 95% CI: 1.12, 8.23) more likely to attain adequate dietary diversity than their counterparts. With regard to market exchange participation, pregnant women who were involved in market exchange participation had 2.5 times (AOR = 2.54, 95% CI: 3.27, 9.83) greater chance of consuming the recommended minimum dietary diversity than those who did not involve in the market.

## 4. Discussion

The study has investigated the dietary diversity and associated factors among pregnant women attending antenatal clinic in Shashemane, Oromia region, Central Ethiopia. The mean DDS of pregnant women was 3.5 ± 2.5 SD, and only one-quarter of pregnant women had adequate dietary diversity and most pregnant women (74.6%) did not receive minimum dietary diversity. The findings of this study are lower than the findings of a study done in Pakistan [19], Bangladesh [20], and Kenya [17]. This difference might be due to the fact that the difference in measurement dietary diversity except Bangladesh, which used 10 food groups, consuming 5 or more food groups are classified as adequate dietary diversity [10]. Other studies used the 14 food groups, and those consumed four or more food groups of the fourteen food groups were classified as minimum dietary diversity which will result in a greater score than this study. Beyond this, sociodemographic, socioeconomic, and seasonal variations and reporting (hence self-reporting) difference might be the possible reason for the discrepancy.

In this study, nearly all (95%) the pregnant mothers had consumed starchy staples (foods made from grain, white roots, tubers and plantains) in the previous 24 h. Conversely, animal products like milk and milk products food group, meat and fish food group, and egg group were least consumed groups. This finding is almost consistent with findings of other studies done in Bangladesh [20], Kenya [17], and Tanzania [21]. This might be due to the fact that the culture of the community, utilization of animal products mainly on the holiday (Christmas and Easter) and annual and marriage ceremonies and lower socioeconomic status to afford animal products having <2000 Ethiopian birr monthly income and limited livestock owning. This implies that women who consume low animal product (meat and dairy products) might be affected by anemia. Hence, not consuming animal products leads to lower adequate dietary diversity, which ends up with risk of anemia. But, this does not always happen because it is affected by different individual factors.

The educational level is the strongest predictor of adequate dietary diversity. It was revealed that increasing the level of maternal education was significantly associated with adequate dietary diversity compared to those mothers who had no formal education. This finding is in line with a study done in Kenya [17], Tanzania [21], and Ghana [22]. The possible explanation for this might be due to the fact that those mothers attaining secondary education and above were more likely to get information regarding nutritional requirement and understand educational messages delivered through different media outlets. For this, as the educational level increases, the level of attaining adequate dietary diversity intake is expected to increase.

The study revealed that as the household income increased, the chances of consuming adequate dietary diversity also increased. Those pregnant women who have higher monthly income were more likely to have adequate dietary diversity than their counterparts. The possible explanation for this might be due to the fact that those women with higher income are associated with increased purchasing power which could be more likely to afford to have diversified foods as compared to pregnant women from a lower household income. This is similar to a study done in Iran [23] and Kenya [17].

Another factor which was found to be associated with an adequate dietary diversity was livestock ownership, which showed that those who had reported owning livestock were more likely to attain a diversified diet. This is supported by a study done in Bangladesh [24]. This might be due to the fact that those women who had reported owning livestock could be more likely to afford easily livestock products without purchasing, and hence, most participants' source of food is through purchasing and also could be more likely to purchase a variety of food by selling their livestock easily, and livestock contain several food groups, which increased diet diversity that may provide micronutrients and macronutrients [25].

Furthermore, women who got husbands' support had greater odds of attaining adequate dietary diversity than those who did not get support from their husband. This is similar to a study done in Bangladesh [20] and Tanzania [21], which states that husbands support by reducing consumption of food away from the home, especially during periods of food shortages. The possible explanation for this could be due to the fact that those women who got support from their husband could share their burden of food and household asset or income shortage and other healthcare-related events, which would decrease individual stress anxiety [26–28] and subsequently leading intake of adequate dietary diversity. Hence, studies confirm that high dietary diversity was associated with a lower level of antenatal stress or anxiety [29–31].

In addition, women who participated in food shopping in the market have greater chance of attaining adequate dietary diversity intake. This finding is supported by studies done in Southern United States [32–34], sub-Saharan Africa [35], Southern Benin [36], and Malawi [37]. This might be due to the fact that those women who participated in the food shopping have higher chance to purchase different foods that are scarce or totally unavailable at home or increase their ability to prepare more diverse foods which provide opportunities to improve their consumption of diverse diet and also support on farm dietary diversity by providing an outlet for household production.

## 5. Limitations

This study cannot make the causal relationship (difficult to know which precedes the exposure or outcome). Hence, it is a cross-sectional study. The study is unable to generalize to the population because of the health-facility nature of this study. In addition, this study might not give the exact figure of the dietary diversity practice due to a recall bias and being self-reported. Beyond this, this study did not address the access and barriers to market access and the extent of partners support.

## 6. Conclusions

This study has shown that the overall consumption of adequate dietary diversity of the pregnant mothers was found to be low. Mother's educational attainment, higher household monthly income, livestock ownership, emotional support by the husband, and market participation were positively associated with the adequate dietary diversity.

In light of this finding, there is a need to support existing and come up with new policies targeting these variables. So, improving the educational status of women, increasing household income, owning of livestock in the household, increasing husbands' support, and improving women's participation in market exchange is recommended to improve women's dietary diversity. Further study should be done on the knowledge-related and behavioral factors of dietary diversity.

## Figures and Tables

**Table 1:** Sociodemographic characteristics of pregnant mothers attending ANC clinics in Shashemane, Oromia, Central Ethiopia, in 2017 (*N*=315).

Variable	Category	Frequency (%)
Age	<25	243 (77.1)
	25–34	64 (20.3)
	35–44	8 (2.5)

Educational status of women	No formal education	23 (7.3)
	Primary education	192 (61)
	Secondary education	52 (16.5)
	College and above	48 (15.2)

Religion	Protestant	225 (71.4)
	Orthodox	42 (13.3)
	Muslim	37 (11.7)
	Others^*∗*^	11 (3.5)

Occupation of women	Employee	72 (22.9)
	Housewife	129 (41.0)
	Merchant	92 (29.2)
	Others^*∗∗*^	22 (7.0)

Husbands' education	No formal education	16 (5.1)
	Primary education	169 (53.7)
	Secondary education	72 (22.9)
	College and above	58 (18.4)

Household income	<2000	252 (80.7)
	2000–3500	37 (11.7)
	>3500	26 (8.3)

Livestock ownership	Yes	148 (47)
	No	167 (53)

Land ownership	Yes	245 (77.8)
	No	105 (22.2)

Household size	1–3	257 (81.6)
	3–6	45 (14.3)
	>6	14 (4.1)

Morbidity in previous 2 weeks	Yes	138 (43.8)
	No	177 (56.2)

Husbands' support	Yes	105 (33.3)
	No	210 (66.7)

Market exchange participation	Yes	272 (86.3)
	No	43 (13.7)

^*∗*^showed catholic, paganism. ^*∗∗*^showed daily laborer, coffee-tea seller.

**Table 2:** Nutritional practices of pregnant mothers attending ANC clinics in Shashemane, Oromia, Central Ethiopia, in 2017 (*N*=315).

Variables		Frequency (%)
Follow specific dietary regimen	Yes	15 (4.8)
	No	302 (95.2)
Use iodized salt	Yes	52 (16.5)
	No	265 (83.5)
Drink coffee or tea	Yes	34 (10.8)
	No	285 (89.2)
Have iron supplement	Yes	26 (8.3)
	No	293 (91.7)
Frequency of meal per day	1–3 times	73 (23.2)
	4 times and above	242 (76.8)
Habits of eating snack	Yes	66 (21.0)
	No	249 (79.0)
Alcohol use in the previous 7 days	Yes	95 (30.2)
	No	220 (99.8)
Main source of food in the household	Own production	142 (45.1)
	Purchasing	165 (52.4)
	Others	8 (2.5)

Others: food aid, getting food as daily laborer from the site they work.

**Table 3:** Dietary diversity frequency of pregnant mothers attending antenatal clinics in Shashemane, Oromia, Central Ethiopia, in 2017 (*N*=315).

Food group consumption in previous 24 h	Frequency (%)
Starch staples (grains, white roots and tuber, and plantains)	299 (95)
Pulses (beans, peas, and lentils)	85 (27)
Nuts and seeds	185 (58.7)
Dairy	105 (33.3)
Meat, poultry, and fish	36 (11.4)
Eggs	11 (3.5)
Dark green leafy vegetables	64 (20.3)
Other vitamin A-rich fruits and vegetables	59 (18.7)
Other vegetables	208 (66)
Other fruits	165 (52.4)
Women dietary diversity	
Adequate	80 (25.4)
Inadequate	235 (74.6)

**Table 4:** Factors associated with dietary diversity of pregnant women attending antenatal clinics in Shashemane town, Oromia, Central Ethiopia, in 2017 (*N*=315).

Variable	Category	Dietary diversity		COR (95% CI)	AOR (95% CI)
		Adequate	Inadequate		
Educational level of women	No formal education	8	15	1	1
	Primary education	20	172	2.15 (0.25, 4.67)	
	Secondary education	17	35	1.78 (0.01, 0.68)	2.31 (2.32, 8.72)^*∗*^
	College and above	35	13	2.5 (2.6, 7.65)	3.18 (1.8, 6.35)^*∗*^

Occupation of wife	Employee	30	42	2.25 (3.52, 10.9)	3.17 (0.63, 7.65)
	Housewife	15	114	1	1
	Merchant	32	60	1.57 (0.68, 5.36)	
	Others	5	17	2.05 (0.17, 9.15)	

Household income	<2000	30	222	1	1
	2000–3500	28	9	2.56 (1.47, 8.26)	2.12 (0.35, 6.58)
	>3500	22	6	1.85 (2.59, 10.2)	2.24 (1.47, 7.78)^*∗*^

Livestock ownership	Yes	60	88	2.31 (1.89, 6.45)	4.15 (2.07, 9.86)^*∗*^
	No	15	152	1	1

Land ownership	Yes	48	197	1	1
	No	32	73	0.05 (0.23, 0.95)	0.4 (0.42, 5.69)

Household size	1–3	66	191	3.15 (1.05, 8.53)	2.75 (0.98, 9.37)
	3–6	10	35	2.43 (0.54, 7.85)	
	>6	4	10	1	1

Morbidity	Yes	53	83	1	1
	No	27	150	0.45 (0.45, 0.94)	0.37 (0.26, 8.35)

Husbands' support	Yes	60	45	2.92 (3.42, 11.6)	3.49 (1.12, 8.23)^*∗*^
	No	20	190	1	1

Shopping	Yes	70	202	2.03 (1.28, 7.98)	2.54 (3.27, 9.83)^*∗*^
	No	10	33	1	1

^*∗*^Statistical significance at *p* value < 0.05 with 95% CI.

## Data Availability

The data used to support the findings of this study are available from the corresponding author upon request.

## Supplementary Materials

Supplementary Materials Supplementary file 1: guidelines for measurement of dietary diversity for pregnant women in Shashemane, Central Ethiopia. Supplementary file 2: STROBE statement—checklist: for cross-sectional study.
