# Supplementary material for: Dietary Diversity and Associated Factors among Pregnant Women Attending Antenatal Clinic in Shashemane, Oromia, Central Ethiopia: A Cross-Sectional Study
Source: J Nutr Metab. 2019 Mar 12;2019:3916864. doi: 10.1155/2019/3916864 (PMC6434279; doi:10.1155/2019/3916864)
Supplement: Supplementary Materials — Supplementary file 1: guidelines for measurement of dietary diversity for pregnant women in Shashemane, Central Ethiopia. Supplementary file 2: STROBE statement—checklist: for cross-sectional study. [file 3916864.f1.docx]

**Supplementary file 1 and 2**

**Supplementary file 1: Guidelines for Measuring Dietary Diversity among Pregnant women attending Antenatal clinic in Shashemane, Oromia, Central Ethiopia, 2017**

| **No** | **group in WMDD Questionarie** | **10 food groups in MDD-W** |
| --- | --- | --- |
| 1  2. | Foods made from Grains  white roots , tubers and and plantains | 1. Grains, white roots and tubers, and plantains |
| 3. | Pulses (beans, peas and lentils) | 2. Pulses (beans, peas and lentils) |
| 4. | Nuts and seeds | 3. Nuts and seeds |
| 5. | milk and milk products | 4. Diary |
| 6.  7.  8. | organ meat  Meat and poultry  fish meats and sea food | 5. meat, poultry and fish |
| 9. | Eggs | 6. Eggs |
| 10. | Dark green leafy vegetables | 7. Dark green leafy vegetables |
| 11.  12. | vitamin A rich vegetables, roots and tubers  Vitamin A rich fruits | 8. Other vitamin-A rich fruits and vegetables |
| 13. | Other vegetables | 9. Other vegetables |
| 14. | other fruits | 10. Other fruits |

**Supplementary file 2: STROBE Statement—Checklist:** Dietary Diversity and Associated Factors among Pregnant women Attending Antenatal clinic in Shashemane, Oromia, Central Ethiopia

| **Section** | | | Item No | | | | Recommendation | | Remark | Page No |
| --- | --- | --- | --- | --- | --- | --- | --- | --- | --- | --- |
| **Title and**  **abstract** | | | 1 | | | | Indicate the study’s design with a commonly used term in the title or the abstract | | **Done** | **Page 1** |
|  |  |  |  |  |  |  | Provide in the abstract an informative and balanced summary of what was done and what was found | | **Done** | **Page 2** |
| Introduction | | | | | | | | |  |  |
| Background/rationale | | | 2 | | | | Explain the scientific background and rationale for the investigation being reported | | **Done** | **Page 3** |
| Objectives | | | 3 | | | | State specific objectives, including any prespecified hypotheses | | **Done** | **Page 3** |
| Methods | | | | | | | | |  |  |
| Study design | 4 | | | Present key elements of study design early in the paper | | | | | **Done** | **Page 4** |
| Setting | 5 | | | Describe the setting, locations, and relevant dates, including periods of recruitment, exposure, follow-up, and data collection | | | | | **Done** | **Page 4** |
| Participants | 6 | | | Give the eligibility criteria, and the sources and methods of selection of participants | | | | | **Done** | **Page 4** |
| Variables | 7 | | | Clearly define all outcomes, exposures, predictors, potential confounders, and effect modifiers. Give diagnostic criteria, if applicable | | | | | **Done** | **Page 5** |
| Data sources/ measurement | 8* | | | For each variable of interest, give sources of data and details of methods of assessment (measurement). Describe comparability of assessment methods if there is more than one group | | | | | **Done** | **Page 5** |
| Bias | 9 | | | Describe any efforts to address potential sources of bias | | | | | **Done** | **Page 5** |
| Study size | 10 | | | Explain how the study size was arrived at | | | | | **Done** | **Page 5** |
| Quantitative variables | 11 | | | Explain how quantitative variables were handled in the analyses. If applicable, describe which groupings were chosen and why | | | | | **Done** | **Page 5** |
| Statistical methods | 12 | | | Describe all statistical methods, including those used to control for confounding | | | | | **Done** | **Page 5** |
|  |  |  |  | Describe any methods used to examine subgroups and interactions | | | | | **Not applicable** |  |
|  |  |  |  | Explain how missing data were addressed | | | | | **Done** | **Page 5** |
|  |  |  |  | If applicable, describe analytical methods taking account of sampling strategy | | | | | **Done** | **Page 5** |
|  |  |  |  | Describe any sensitivity analyses | | | | | **Notapplicable** |  |
| Results | | | | | | | | |  |  |
| Participants | | 13* | | | Report numbers of individuals at each stage of study—eg numbers potentially eligible, examined for eligibility, confirmed eligible, included in the study, completing follow-up, and analysed | | | | **Done** | **Page 6** |
|  |  |  |  |  | Give reasons for non-participation at each stage | | | | **Done** | **Page 6** |
|  |  |  |  |  | Consider use of a flow diagram | | | | **Not applicable** |  |
| Descriptive data | | 14* | | | Give characteristics of study participants (eg demographic, clinical, social) and information on exposures and potential confounders | | | | **Done** | **Page 6** |
|  |  |  |  |  | Indicate number of participants with missing data for each variable of interest | | | | **Done** | **Page 6** |
| Outcome data | | 15* | | | Report numbers of outcome events or summary measures | | | | **Done** |  |
| Main results | | 16 | | | Give unadjusted estimates and, if applicable, confounder-adjusted estimates and their precision (eg, 95% confidence interval). Make clear which confounders were adjusted for and why they were included | | | | **Done** | **Page 6 & 7** |
|  |  |  |  |  | Report category boundaries when continuous variables were categorized | | | | **Done** | **Page 7, 16 &**  **17** |
|  |  |  |  |  | If relevant, consider translating estimates of relative risk into absolute risk for a meaningful time period | | | | **Not applicable** |  |
| Other analyses | | 17 | | | Report other analyses done—eg analyses of subgroups and interactions, and sensitivity analyses | | | | **Not applicable** |  |
| Discussion | | | | | | | | |  |  |
| Key results | | | | | | 18 | | Summarise key results with reference to study objectives | **Done** | **Page 8 and 9** |
| Limitations | | | | | | 19 | | Discuss limitations of the study, taking into account sources of potential bias or imprecision. Discuss both direction and magnitude of any potential bias | **Done** | **Page 10** |
| Interpretation | | | | | | 20 | | Give a cautious overall interpretation of results considering objectives, limitations, multiplicity of analyses, results from similar studies, and other relevant evidence | **Done** | **Page 8 and 9** |
| Generalizability | | | | | | 21 | | Discuss the generalizability (external validity) of the study results | **Done** | **Page 10** |
| Other information | | | | | | | | |  |  |
| Funding | | | | | | 22 | | Give the source of funding and the role of the funders for the present study and, if applicable, for the original study on which the present article is based | **Not applicable** |  |
